# Reconceptualizing dermatology patient care and education during the COVID-19 pandemic and beyond

**DOI:** 10.1016/j.ijwd.2021.10.010

**Published:** 2021-10-28

**Authors:** Shari R. Lipner, Shweta Shukla, Claire R. Stewart, Sara Behbahani

**Affiliations:** a Department of Dermatology, Weill Cornell Medicine, New York, New York; b SUNY Downstate Medical Center, Brooklyn, New York; c Weill Cornell Medical College, New York, New York; d Rutgers New Jersey Medical School, Newark, New Jersey

**Keywords:** COVID-19, SARS-CoV-2, academic dermatology, medical education, dermatology education, resident education, medical student education, virtual learning, e-learning

**What is known about this subject in regard to women and their families?**

• The virtual didactic curriculum and telemedicine, propelled by the COVID-19 pandemic, have been helpful for the continuity of dermatologic education and patient care.
• Live teaching sessions are essential for the education of physicians who serve female patients and their families.
• In-person visits are necessary for the dermatologic care of female patients and their families.

**What is new from this article as messages for women and their families?**

• Reconceptualizing the dermatology curriculum and teachings would positively affect the education of physicians who serve female patients and their families.
• Expanding dermatology patient care to nontraditional hours and days would be beneficial for female patients and their families.

Alt-text: Unlabelled box

The COVID-19 pandemic has transformed dermatology clinical practice and education. A multimodal approach has been suggested to support clinical practice and medical education, including virtual didactics, teledermatology, and self-directed learning using online platforms (Muftiet al., 2020). Virtual platforms have been essential for the continuity of patient care and medical student, resident, and attending education during the pandemic (Cook and Steinert, 2013; Stewart and Lipner, 2021). As vaccination efforts continue, we suggest adjunctive and alternative approaches, including revamping in-person clinical care and standardizing didactics across residency programs with a blended/hybrid model, with long-lasting benefits even after the pandemic (Table 1).

**Table 1:** Proposed methods for expansion of in-person patient care and dermatology education

Challenge	Solution	Pros	Cons
Inadequate in-person patient interactions	• Expansion of clinic hours • Partnerships with local community hospitals • Partnerships with private practice dermatologists	• Higher patient volume if there is demand • Nontraditional clinic hours may be beneficial to the female patients and their families • Increased diversity of preceptors • Increase resident and faculty (full-time and voluntary) engagement	• Increased nursing and administrative costs • Potential for physician burnout • Long and arduous credentialing processes
Live educational sessions	• Prerecorded didactics, followed by small, in-person discussion groups and journal clubs • Cadaver laboratories with limited numbers of residents	• Decreases overall time burden on faculty and residents as in-person sessions are limited to discussions • Provides meaningful and focused interactions	• Preference for live didactics by residents and/or faculty • Insufficient faculty to run smaller sessions • Increased time burden on faculty
Mentorship	• Partnerships with local dermatologic societies to facilitate attending/trainee interactions	• Provides opportunities for increased interactions with coresidents and senior faculty	• May be time consuming for residents and attendings who already have clinical responsibilities

Telemedicine has revolutionized the delivery of patient care (Muftiet al., 2020), but in-person visits are necessary for the diagnosis of some dermatologic conditions, full body-skin examinations, and procedures. Vaccination efforts have increased in-person patient volume in some areas, but volumes are still low in other locations due to COVID-19-induced limitations (Comeret al., 2020). We suggest several solutions to increase live patient visit volumes, as well as for programs to be evaluated and implemented based on individual needs and resources. For example, where feasible, expanding clinic hours to early mornings, evenings, and/or weekends would maximize patient volume and trainee educational opportunities. Expanding dermatology patient care to nontraditional hours and days could be beneficial for female patients and their families. Private practice dermatologists may provide additional opportunities for resident education, including cosmetics and procedures. While credentialing would take significant time and effort, expanding affiliations with nearby hospitals without dermatologists would provide residents with more opportunities for direct patient contact (Glazer and Rigel, 2017).

Online learning options are beneficial in providing access to educational content to facilitate academic development without time/place constraints and allow for trainees to learn at their own pace. By recruiting dermatologists with expertise in niche fields (i.e., nails, dermoscopy, and bullous diseases) and prerecording lectures, dermatology curriculums could be standardized across residency programs, compensating for deficiencies in programs without experts. Videos demonstrating techniques, including nail matrix biopsies and marsupialization, would be particularly helpful. A similar curriculum could be developed for medical students interested in dermatology (Cook and Steinert, 2013).

By prerecording lectures, dermatology faculty would have more time to hold discussion sections for smaller live trainee groups, which would provide the interactivity that is lacking from recorded sessions (Cook and Steinert, 2013). Instead of multiple faculty members instructing all residents simultaneously in cadaver laboratories for procedural training, each attending could instruct smaller groups. If faculty are limited, graduating residents and those requiring specific procedural training due to career goals (nail biopsies and platelet-rich plasma for nail and hair specialists, respectively) could be prioritized for hands-on sessions. Moreover, trainings, journal clubs, and lectures could resume in smaller, in-person groups to increase engagement, interest, and participation (Cook and Steinert, 2013). Local dermatology societies could establish mentorship programs to facilitate attending/trainee interactions.

The COVID-19 pandemic has provided an impetus for educators to rethink medical education and find innovative ways to deliver dermatologic patient care and instruction. Virtual platforms may enhance academic dermatology needs in some ways, but in-person interactions for patients, faculty, and trainees are vital for patient care and education. Our suggestions may support academic dermatology departments in cross-collaborating and sharing best practices to provide outstanding clinical care and enhancing dermatology education.
